# A Novel Germline *MLH1* In-Frame Deletion in a Slovenian Lynch Syndrome Family Associated with Uncommon Isolated PMS2 Loss in Tumor Tissue

**DOI:** 10.3390/genes11030325

**Published:** 2020-03-18

**Authors:** Gašper Klančar, Ana Blatnik, Vita Šetrajčič Dragoš, Vesna Vogrič, Vida Stegel, Olga Blatnik, Primož Drev, Barbara Gazič, Mateja Krajc, Srdjan Novaković

**Affiliations:** 1 Department of Molecular Diagnostics, Institute of Oncology Ljubljana, SI-1000 Ljubljana, Slovenia; gklancar@onko-i.si (G.K.); vsetrajcic@onko-i.si (V.Š.D.); vvogric@onko-i.si (V.V.); vstegel@onko-i.si (V.S.); 2 Cancer Genetics Clinic, Institute of Oncology Ljubljana, SI-1000 Ljubljana, Slovenia; ablatnik@onko-i.si (A.B.); mkrajc@onko-i.si (M.K.); 3 Department of Pathology, Institute of Oncology Ljubljana, SI-1000 Ljubljana, Slovenia; oblatnik@onko-i.si (O.B.); pdrev@onko-i.si (P.D.); bgazic@onko-i.si (B.G.)

**Keywords:** Lynch syndrome, MMR, novel *MLH1* variant, segregation analysis, isolated PMS2 loss

## Abstract

The diagnostics of Lynch syndrome (LS) is focused on the detection of DNA mismatch repair (MMR) system deficiency. MMR deficiency can be detected on tumor tissue by microsatellite instability (MSI) using molecular genetic test or by loss of expression of one of the four proteins (MLH1, MSH2, MSH6, and PMS2) involved in the MMR system using immunohistochemistry (IHC) staining. According to the National Comprehensive Cancer Network (NCCN) guidelines, definitive diagnosis of LS requires the identification of the germline pathogenic variant in one of the MMR genes. In the report, we are presenting interesting novel *MLH1* in-frame deletion LRG_216t1:c.2236_2247delCTGCCTGATCTA p.(Leu746_Leu749del) associated with LS. The variant appears to be associated with uncommon isolated loss of PMS2 immunohistochemistry protein staining (expression) in tumor tissue instead of MLH1 and PMS2 protein loss, which is commonly seen with pathogenic variants in *MLH1*. The variant was classified as likely pathogenic, based on segregation analysis and molecular characterization of blood and tumor samples. According to the American College of Medical Genetics (ACMG) guidelines, the following evidence categories of PM1, PM2, PM4, and PP1 moderate have been used for classification of the novel variant. By detecting and classifying the novel *MLH1* variant as likely pathogenic, we confirmed the LS in this family.

## 1. Introduction

According to the European Network of Cancer Registries, colorectal cancer (CRC) is the second leading cause of cancer death in men and the third in women in Europe [1]. 

The most common genetic susceptibility for CRC is Lynch syndrome (LS)—also being one of the most common hereditary cancer syndrome [2]. Patients with LS have markedly increased lifetime risk of CRC and endometrial cancer (EC), as well as ovarian, gastric, hepatobiliary, bladder, renal, brain, breast, prostate, small intestine, pancreatic and sebaceous skin cancers [3,4,5,6,7]. The penetrance of LS is approximately 52% for CRC, 57% for EC, 38% for ovarian, and less than 20% for other previously mentioned cancers [8]. Early detection of LS is therefore of great importance, since prophylactic surgery may effectively prevent endometrial and ovarian cancer [9,10] while colonoscopy surveillance has been proven to reduce morbidity and mortality in CRC by 65% to 70% [11,12,13]. According to the National Comprehensive Cancer Network (NCCN) guidelines, the diagnosis of LS based on clinical criteria is suboptimal—consequently, a definitive diagnosis requires the identification of the pathogenic variant (PV) in one of LS associated genes [7,8,14,15]. 

LS is an autosomal dominant genetically heterogeneous disorder mostly caused by germline PVs in *MLH1*, *MSH2*, *MSH6*, and *PMS2*—the principle genes in the DNA mismatch repair (MMR) system [16,17]. In minority, LS can also be caused by germline PVs in *MLH3* or *MSH3* genes [18]. Germline *MLH1* and *MSH2* PV carriers have significantly higher lifetime cancer risks for any LS associated cancers compared to carriers of *MSH6* and *PMS2* variants, reflecting functional redundancy of MSH6 and PMS2 [19,20]. There is a large number of unique PVs associated with LS that are distributed throughout all coding regions of the MMR genes [14,16,21]. The majority of LS-associated variants are loss of function variants (e.g., stop codon or frameshift variants) causing protein truncation. Variants leading to the change of an amino-acid (AA) residue in a highly conserved region are in general also considered pathogenic [21,22]. PVs in MMR genes result in the accumulation of replication errors that are preferentially accumulated in microsatellites regions of the genome [23]. This molecular phenotype is known as microsatellite instability (MSI) and is detected in a high percentage of LS tumors [24]. Additionally, immunohistochemical (IHC) staining for MMR proteins will show a characteristic pattern of protein expression depending on the underlying MMR gene PV [8,25].

Non-truncating variants (e.g., missense, in-frame variants) can be very difficult to interpret. These genetic alterations lead to the production of proteins with various degrees of functionality, ranging from having no effect on protein to highly affecting protein, which highly increases cancer risk [19,26]. The determination of clinical significance often involves a lot of effort and depends on the nature of the alteration itself (e.g., location in transcript, conservation status, associated biochemical consequences), variant’s co-segregation with disease/phenotype (e.g., family size and history of cancer, availability of blood and tissue samples), data from various functional assays and detailed clinical data [19,26].

Here, we report a novel *MLH1* in-frame deletion LRG_216t1:c.2236_2247delCTGCCTGATCTA p.(Leu746_Leu749del) associated with LS, classified as likely pathogenic based on segregation analysis and molecular characterization of blood and tumors. Interestingly, the variant appears to be associated with uncommon isolated loss of PMS2 protein expression in tumor tissue instead of MLH1 and PMS2 loss, which is usually seen with PVs in *MLH1* [27,28].

## 2. Materials and Methods 

All living participants underwent genetic counselling before testing, in accordance with our clinical pathways and currently valid guidelines. Genetic counselling and testing at our institution has always been performed in accordance with the provisions of the Oviedo Convention. All living participants gave their informed consent for inclusion before they participated in the study. The study was conducted in accordance with the Declaration of Helsinki. Genetic testing on the tissue of the deceased relatives was performed in accordance with the provisions of the National Medical Ethics Committee’s approval (138/05/11, date of approval: 28.5.2011)

All diagnosis of cancers were verified in the Cancer Registry of Republic of Slovenia (http://www.slora.si/en). 

### 2.1. Participants

The proband (III-1) was first seen at our Cancer Genetics Clinic with the diagnosis of carcinoma of the caecum at the age of 39. He had undergone a right hemicolectomy with a L-L anastomosis. Histopathological examination revealed a poorly differentiated adenocarcinoma (stage pT3N2M0). He received postoperative chemotherapy with capecitabine and oxaliplatin and is currently disease-free. His personal history was otherwise unremarkable. 

**Figure 1:**
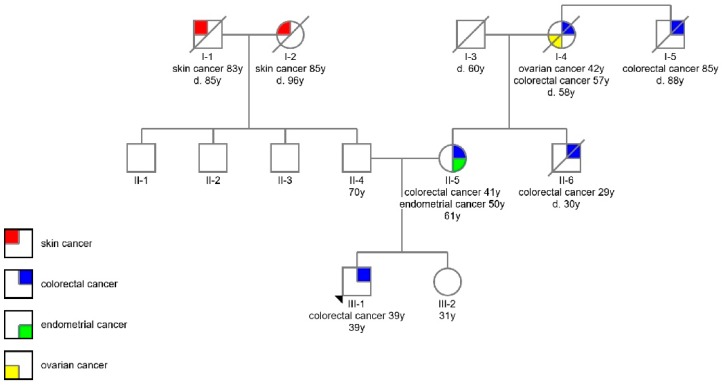
The genealogies including age and cancer types.

The proband’s mother (II-5) developed adenocarcinoma of the ileal flexure aged 41 and an EC aged 50. Her brother (II-6) (proband’s uncle) developed a carcinoma of the caecum aged 29 and died a year later, at the age of 30. Her mother (I-4) (proband’s grandmother) had been treated for ovarian cancer at the age of 43 and carcinoma of the caecum aged 57. She died at the age of 58. The grandmother’s brother (I-5) (proband’s great uncle) developed a rectal carcinoma at the advanced age of 85. The proband’s only sibling (III-2), his sister, was healthy at the age of 31. On the proband’s father’s side of the family only squamous cell skin cancers were reported. The genealogies including age and cancer types are summarized in Figure 1.

Based on proband’s (III-1) personal and family history (early age of onset, right-sided disease, EC and ovarian carcinoma affecting female relatives), LS was seen as the most likely diagnosis in our proband. He was therefore offered genetic testing. Subsequently, his mother (II-5) and sister (III-2) were also tested, and additional germline genetic testing was performed from formalin-fixed paraffin embedded (FFPE) tissue samples from his uncle (II-6) and great uncle (I-5). In addition, IHC staining was performed on FFPE tumor tissue samples from the proband (III-1), his mother (II-5), uncle (II-6) and great uncle (I-5). The samples were also tested for MSI. Tumor mutational burden (TMB) was assessed in the proband’s (III-1) tumor sample.

### 2.2. Immunohistochemistry 

Immunohistochemistry (IHC) staining of non-tumor- and tumor-FFPE samples for MMR proteins MLH1, MSH2, MSH6, and PMS2 was performed according to established laboratory protocol at the Department of Pathology at the Institute of Oncology Ljubljana, Slovenia. Briefly, IHC staining was performed on 2 to 4 µm FFPE tissue sections using fully automated IHC system Ventana Benchmark XT (Ventana Roche, Oro Valley, USA). For this purpose, commercially available monoclonal antibodies for MLH1 (clone ES05, #M3640) (DAKO Agilent, Santa Clara, USA), PMS2 (clone EP51, #M3647) (DAKO Agilent), MSH2 (clone G219-1129, #556349) (BD Pharmingen, Franklin Lakes, USA) and MSH6 (clone SP93, #287R) (CellMarque, Rocklin, USA) were used. Primary antibodies were visualized using a three–step multimer detection system OptiView DAB IHC Detection Kit (Ventana Roche) following the manufacturer's protocol. MLH1 and PMS2 detection was enhanced using OptiVew Amplification Kit (Ventana Roche). All protocols passed external quality assessment in NEQAS and NORDIQC. Normal appendix and colon carcinomas with confirmed protein expression or loss of protein expression were used as controls. 

### 2.3. DNA Isolation

Genomic DNA was isolated according to established laboratory protocols from a whole blood sample using InnuPREP Master Blood Kit (Analytik Jena, Jena, Germany), and from FFPE tissue sample using QIAamp DNA FFPE Tissue Kit (Qiagen, Hilden, Germany). 

### 2.4. Microsatellite Instability

Paired blood or non-tumor- and tumor-FFPE tissue samples were used for the evaluation of microsatellite instability (MSI) applying fluorescent multiplex PCR reaction of six mononucleotide repeat markers: BAT25, BAT26, BAT40, NR21, NR22, and NR27, and one polymorphic dinucleotide marker as internal control D3S1260, according to established laboratory protocol. For interpretation, MSI at ≥3 markers was defined as MSI-high (MSI-H), instability at one or two markers was defined as MSI-low (MSI-L), and no instability at any of the tested markers was defined as MSI-stable (MSI-S). The method was summarized by Buisine and colleagues [29]. Fragments were separated with capillary gel electrophoresis on ABI Genetic Analyzer 3500 and fragment analysis was performed using GeneMapper Software version 4.0 (both Applied Biosystems, Foster City, USA).

### 2.5. Next Generation Sequencing

#### 2.5.1. Genotyping for Germline Alterations

Blood DNA samples for targeted next generation sequencing (NGS) were prepared following the manufacturer’s protocol using Nextera DNA Library Preparation Kit in combination with TruSight Cancer Panel (Illumina, San Diego, USA) to enrich all exon regions and exon/intron boundaries ± 25 nucleotides of *MLH1*, *MSH2*, *MSH6,* and *PMS2*, and 90 other genes. The sample was sequenced on Illumina MiSeqDx Sequencing System with MiSeq Reagent kit v2 (Illumina) following the manufacturer’s protocol and recommendations for quality control. Samples were analyzed according to the established laboratory protocol [30]. The presence of copy number variations (CNVs) in *MLH1*, *MSH2*, *MSH6,* and *PMS2* was analyzed using SeqNext v4.4.0 (JSI medical systems, Ettenheim, Germany). Due to pseudogenes in *PMS2,* the CNV analysis was uncertain, and additional MLPA testing was therefore made.

#### 2.5.2. Genotyping for Sporadic Alterations

Selected tumor DNA sample for NGS were prepared following the manufacturer’s protocol using TruSight Tumor 170 (Illumina). Samples were sequenced on Illumina NextSeq 550 Sequencing System with NextSeq Reagent kit v3 (Illumina) following the manufacturer’s protocol and recommendations for quality control. Read alignment to the hg19 reference genome and variant calling was performed using TruSight Tumor 170 v2 Local App software (Illumina). Variant annotation was performed using Variant Studio 3.0 software and Alamut Visual v2.14 software. Tumor mutational burden was defined as the number of somatic variants in coding regions, base substitution, and small indels (insertions and deletions less than 20 nucleotides) per megabase (mut/Mb) of gene panel [31,32]. 

### 2.6. MLPA

Due to pseudogenes in *PMS2,* CNVs were additionally tested with Multiplex Ligation dependent Probe Amplification (MLPA). SALSA MLPA P008 PMS2 Probemix was performed following the manufacturer’s protocol including recommendations for quality control parameters (MRC-Holland, Amsterdam, Netherlands).

### 2.7. Sanger Sequencing

Direct Sanger sequencing was performed to confirm the presence of novel *MLH1* mutation in tested members of the LS family. Collected samples were bidirectionally sequenced according to the established laboratory protocol, using in-house designed primers. Primers are available upon request. Amplicons were sequenced using BigDye Terminator v3.1 Sequencing Kit and ABI Genetic Analyzer 3500 (both Applied Biosystems). 

## 3. Results

**Figure 2:**
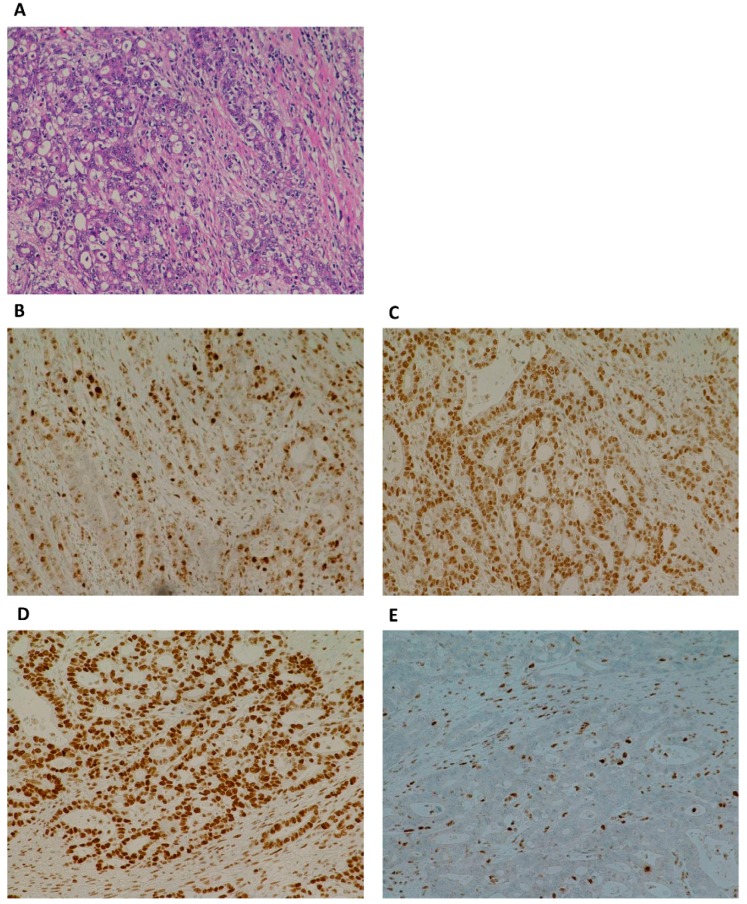
Pathohistological features of proband’s (III-1) tumor; adenocarcinoma of the cecum. (**A**) Histological analysis (hematoxylin and eosin) revealed a poorly differentiated adenocarcinoma of the cecum, consisting of poorly formed glands and sheets of cells with large vesicular nuclei and prominent nucleoli. (**B–E**) Immunohistochemical staining of tumor cells showed retained expression of MLH1 (**B**), MSH2 (**C**) and MSH6 (**D**), and loss of expression (staining) of PMS2 (**E**). Note that PMS2 staining was lost in tumor cells, while internal control (stromal cells, immune cells) have intact PMS2 expression.

**Figure 3:**
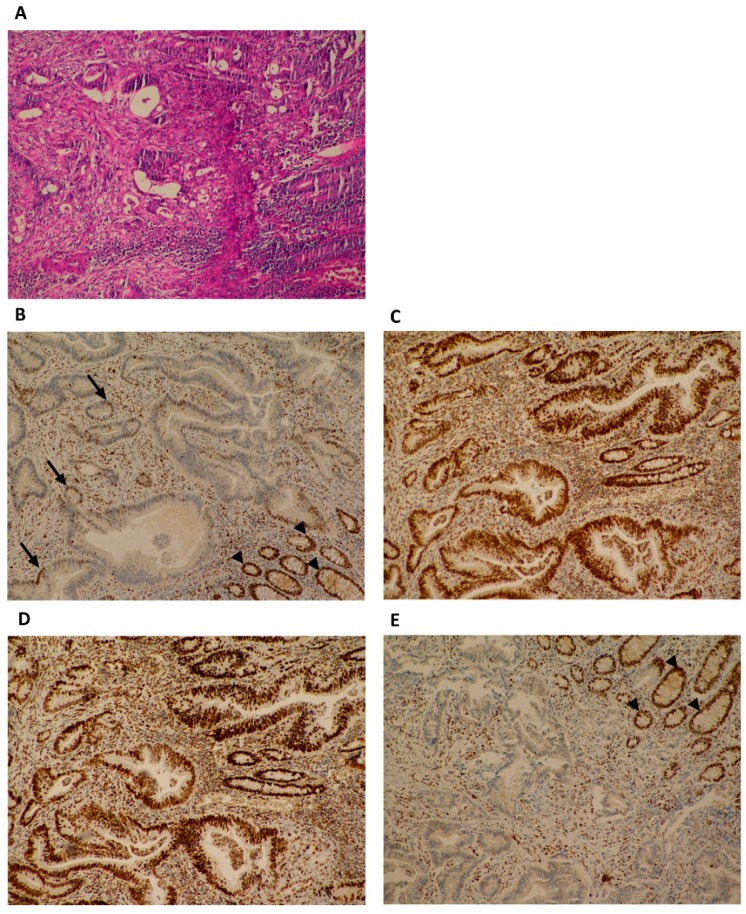
Pathohistological features of proband’s mother’s (II-5) tumor; adenocarcinoma of the ileal flexure. (**A**) Histological analysis (hematoxylin and eosin) revealed a moderately differentiated adenocarcinoma of the ileal flexure. (**B–E**) Immunohistochemical staining of tumor cells showed retained expression of MLH1 (in approximately 5% of tumor cell nuclei; black arrows) (**B**), MSH2 (**C**) and MSH6 (**D**), and loss of expression of PMS2 (**E**). Note that nuclei of normal adjacent epithelium (arrowheads on panel B and E) have an intact expression.

A novel in frame deletion in the *MLH1* gene LRG_216t1:c.2236_2247delCTGCCTGATCTA p.(Leu746_Leu749del) was detected in peripheral blood of our proband (III-1) and his mother (II-5) with NGS sequencing. The variant was later confirmed by Sanger sequencing in proband’s blood as well as in his tumor tissue (Figure S1). The variant was submitted to NCBI ClinVar in July 2019 under accession number VCV000638664.1. This variant has not been previously reported in specific literature or in the public databases (NCBI ClinVar, Qiagen HGMD Professional, LOVD and InSiGHT). The variant is rare, as it was not encountered in control populations (gnomAD, ExAC, 1000 Genomes Project, NHLBI ESP) nor in our cohort of more than 3000 individuals who underwent germline testing. No additional germline PV in *MLH1*, *MSH2*, *MSH6,* or *PMS2* genes was detected by NGS or MLPA analysis. In the *PMS2* gene, only clearly benign variants have been detected, which are listed in Table S1. Even though we have not detected a PV in *PMS2* gene, the IHC staining of proband’s tumor tissue showed an isolated loss of the PMS2 protein, but not MLH1—as expected (Figure 2, Figure 3, Figures S2 and S3). 

To clarify the pathogenicity of the newly discovered *MLH1* variant, the segregation of the variant in the family was conducted from family members’ blood or FFPE-tissue samples (Table 1). The proband’s mother (II-5) carried the same variant in blood and tumor-FFPE samples. For his uncle (II-6) and his great uncle (I-5) we only had access to FFPE samples, and only his uncle (II-6) was a carrier of the variant. Unfortunately, FFPE samples from proband’s grandmother (I-4) were not available. His sister (III-2) was not a carrier of the variant. Full detailed information is shown in Figure 1 and Table 1.

**Figure 4:**
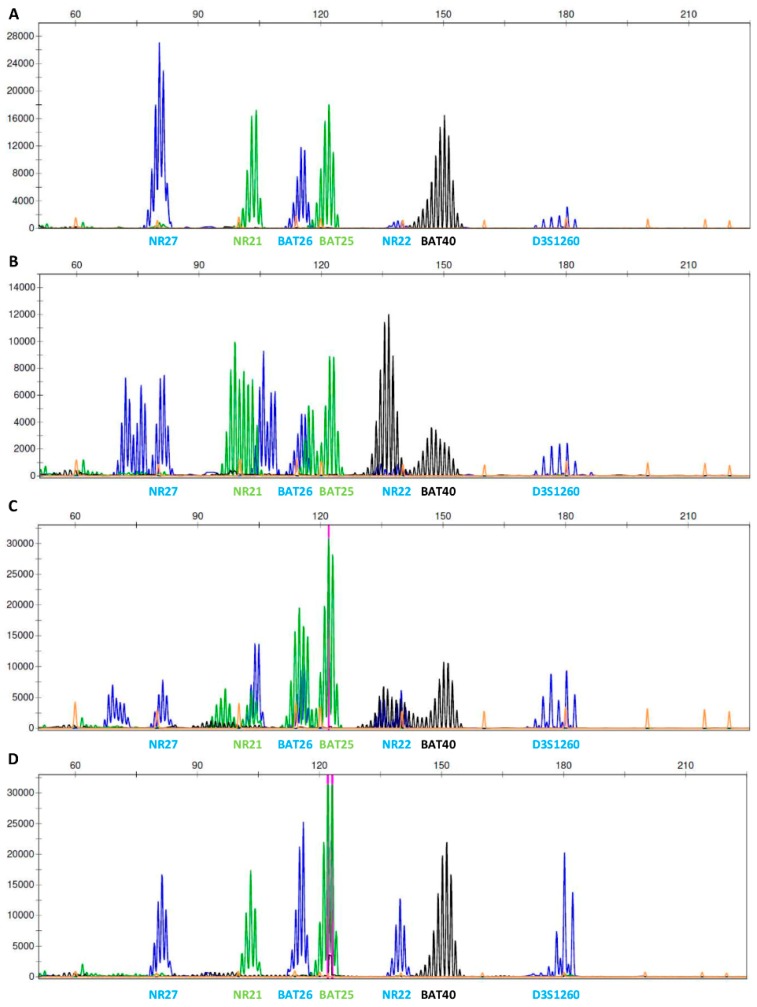
Microsatellite instability (MSI) of proband’s (III-1) tumor sample; adenocarcinoma of the cecum, detected by fragment analysis. (**A**) Negative control sample. (**B)** Positive control sample. (**C**) Proband’s tumor sample with MSI-high status. (**D)** Proband’s non-tumor (blood) sample with MSI-stable status. Note, we detected six mononucleotide repeat markers: BAT25, BAT26, BAT40, NR21, NR22, and NR27, and one polymorphic dinucleotide marker as internal control D3S1260.

Furthermore, the expression of MMR proteins by IHC and MSI status was assessed from non-tumor and tumor-FFPE samples of the family members, according to their availability (Table 1). All carrier’s tumor samples (III-1, II-5, and II-6) lacked the expression of PMS2 protein and had MSI-H status (Table 1, Figure 2, Figure 3, Figure 4, Figures S2 and S3). Immunohistochemical staining of tumor cells was inadequate for assessment of MLH1 expression in tumor samples of EC of proband’s mother (II-5) since MLH1 staining does not show any immunoreactivity in tumor cells nor in internal control (stromal cells, immune cells) (Figure S2). In her adenocarcinoma of the ileal flexure, MLH1 protein expression was 5% (Figure 3). Non-carriers of the variant (III-2 and I-5) had normal expression of all MMR proteins (MLH1, MSH2, MSH6, and PMS2), and MSI-S status. 

Additionally, the TMB of proband’s tumor (III-1) was 22 mutations per Mb (Table 1) (determined by NGS).

According to the ACMG guidelines the *MLH1* variant LRG_216t1:c.2236_2247delCTGCCTGATCTA p.(Leu746_Leu749del) was classified as likely pathogenic [33]. The following ACMG classifying criteria were used: PM1, PM2, PM4, and PP1 moderate. 

## 4. Discussion

Patients with a dysfunctional mismatch repair (MMR) system are at high risk for developing CRC [8]. Thus, a timely and accurate diagnosis is important in order to implement an appropriate program of surveillance, which improves long-term survival in individuals carrying a MMR gene defect.

The functional MMR system is vital to maintain genomic stability by correcting single-base mismatches and insertion/deletion loops arising during replication [19,34]. Dysfunction of MMR proteins results in mutated phenotype and MSI of tumors associated with LS. MSI-high phenotype detected by PCR indicates there is a defect in the MMR system, but cannot predict which gene is involved in pathogenesis. Conversely, loss of protein expression detected by IHC in MMR proteins can guide genetic testing to a particular protein or protein complex [7]. In MMR system, two mismatch-binding complexes are crucial. The first complex MutSα (MSH2 and MSH6) has a role in recognition and second complex MutLα (MLH1 and PMS2) has a role in the repair of single-base mismatches and insertion/deletion loops [19,35]. 

The heterodimer pairing of MMR proteins holds the key to interpreting the results from IHC. MLH1 forms a complex with PMS2 and is responsible for complex stability [36,37]. Therefore, isolated loss of PMS2 protein indicates a defect in *PMS2* gene, while a defect in *MLH1* gene results in loss of expression of both MLH1 and PMS2 proteins [38]. In our study, tumor-FFPE samples of the patients with LS associated cancers had a MSI-H phenotype and positive IHC staining (expression) for MLH1, MSH2, and MSH6, but negative expression for PMS2 (Table 1). However, in the patient II-5, only 5 % of tumor nuclei had retained expression of MLH1 protein.

Based on IHC staining of our cases, one would expect the detection of a germline PV in *PMS2* as loss of PMS2 expression is known to be associated with germline or somatic PV as well as epigenetic inactivation of *PMS2* promotor region [39]. Surprisingly, DNA testing revealed no PV variants in *PMS2* gene, yet showed a novel germline *MLH1* variant LRG_216t1:c.2236_2247delCTGCCTGATCTA p.(Leu746_Leu749del). Moreover, the high penetrance and early-onset disease seen in our family is more in line with what one would expect in *MLH1*-associated LS than *PMS2*-associated LS. 

The novel variant LRG_216t1:c.2236_2247delCTGCCTGATCTA p.(Leu746_Leu749del) is a 12 bp deletion maintaining the reading frame by deleting 4 AAs from the C-terminal homology domain region (CTH) (AA residues 500-756) of the ultimate exon 19 of the *MLH1* [36,40]. We hypothesize that the deleted part of the MLH1 protein is responsible for the loss of heterodimerization with PMS2 and therefore isolated loss of PMS2. Reported deletion in *MLH1* is located in the region crucial for MLH1-PMS2 complex assembly and subsequently the loss of staining with PMS2 is the result of this deletion. Hinrichsen et al. [41] have shown that PMS2 degradation was phosphorylation-dependent and that treatment with a phosphatase inhibitor led to PMS2 degradation when MLH1 C-terminal domain was truncated or if the part of MLH1 protein essential for interaction with PMS2 was lacking. We therefore assume that in our cases the MLH1-PMS2 protein complex could not form, which led to degradation of PMS2 and loss of immunoreactivity for PMS2. Different studies in CRC and EC tumors have discovered that *MLH1* PVs can lead to loss of PMS2 expression, while MLH1 expression is preserved [27,42,43]. Buermeyer et al. [36] demonstrated that truncation of the CTH reduces the stability of heterodimer, although mutant MLH1 protein retained significant capacity for interaction with PMS2. They concluded that CTH domain is important for interaction of MLH1 with PMS2 (dimerization) and stabilization of MutLα complex [36,40]. Thus, CTH is necessary for error correction, checkpoint signaling and promoting interaction with, and the stability of PMS2 [36]. CTH domain is also highly conserved among different species, such as mammalians, insects, plants, fungi, and worms [36]. In general, germline PVs in *MLH1* have been identified along the entire length of the gene and are frequently located in the ATP binding domain and CTH [19,44]. In a report by Silva et al. [45], two of four cases with germline PV in *MLH1* whose tumors showed isolated loss of PMS2 expression had truncating PV in exons 16 and 17 and involving the CTH domain. Deletions resulting in C-terminal truncation of MLH1 with retained immunoreactivity have been reported earlier [46,47], cautioning genetic counselors to search for *MLH1* mutations in cases of isolated PMS2 loss when family history is highly suspicious for *MLH1,* and not a *PMS2* defect. Additionally, a recent paper by Hechtman et al. [48], showed an enrichment of deleterious missense variants over truncating variants in immunohistochemically discordant, MSI-H cases mostly involving *MLH1*.

However, the novel *MLH1* variant detected in our proband was one of those variants that were difficult to interpret, due to its non-truncating effect and its location in the ultimate exon of the gene. Variants in the last exon must be cautiously classified because there is a higher probability that the transcript escapes nonsense-mediated decay. In our case, the protein length changes, due to in-frame deletion of 4 AAs, which is more likely to disrupt protein function compared to missense changes. In general, larger length changes encompassing more conserved AA residues are more suggestive of pathogenicity. Thus, these variants are difficult to interpret without functional assay [33].

Since the detected variant has not been previously reported in LS families, segregation analysis was performed to additionally support the clinical classification. There is a clear co-segregation of the novel *MLH1* variant with LS associated cancers in multiple affected family members (Table 1 and Figure 1). Three of tested family members (III-1, II-5, and II-6) with LS associated cancers were carriers of the novel variant (Table 1). Lower allele fraction (20 %) of *MLH1* variant was detected in the sample of lymph node nearby colon of the carrier II-5. We assume it is a consequence of low-quality DNA, extracted from FFPE tissue sample. Unfortunately, there were no FFPE samples available for proband’s grandmother (I-4). Since she died quite early at the age of 58 and was diagnosed with ovarian cancer at the age of 42, and CRC at the age of 57, it could be speculated that she was a carrier of the variant causative for LS in her family. Proband’s sister (III-2) was healthy when she was tested and had no clinical manifestation of LS. Proband’s great uncle (I-5) had a rectal cancer at the age of 86, making it unlikely that his disease was the result of LS, but more likely of a somatic origin. 

## 5. Conclusions

In this report, we present a novel and previously unreported likely pathogenic *MLH1* in-frame deletion LRG_216t1:c.2236_2247delCTGCCTGATCTA p.(Leu746_Leu749del). Using ACMG guidelines [33], the variant was classified as likely pathogenic, applying evidence categories PM1, PM2, PM4, and PP1 moderate. PM1 was used considering the deletion location in a mutational hot spot and critical and well-established functional CTH domain. Several PVs affecting CTH domain have been identified indicating pathogenic effects of variants in this domain [26,36,49,50]. However, there are no clear-cut recommendations or hotspot region databases to address PM1 criterion. According to NCBI ClinVar (access date: June 27, 2019) in the region of novel PV (exon 19), 52 pathogenic alterations are reported out of 70 classified variants (variants of uncertain clinical significance were not included), indicating high percentage of PVs (74.2%) in this domain. PM2 was used due to the absence of the variant from control populations in ESP, 1000 Genomes Project, ExAC and gnomAD. PM4 was used considering the change (loss of 4 AAs) in protein length as a result of an in-frame deletion in a non-repeat region. PP1-moderate was used due to variant co-segregation with disease in multiple affected family members. Proven variant co-segregation with affected family members emphasizes the importance of linking molecular and clinical data. A functional study (e.g., yeast two-hybrid assay [51,52] will be needed to conclusively determine the effect of this variant on the interaction of MLH1 and PMS2, and the stabilization of the MutLα complex. This report contributes to the characterization of PV spectra in *MLH1* leading to LS.

## Figures and Tables

**Table 1:** Testing for Lynch syndrome: characterization and segregation analysis in available family members.

Family Member	Tumor as a Result of *MLH1* Variant Causing Lynch Syndrome	Material		Tumor Cells (%)	IHC (Expression)				MSI Status	*MLH1* Variant Allele Fraction (%)	MLPA (*PMS2* Gene)	TMB (mut/Mb)
		Tumor Sample	Non-Tumor Sample		MLH1 (%)	MSH2 (%)	MSH6 (%)	PMS2 (%)				
Proband III-1	yes	/	blood	0	N/A	N/A	N/A	N/A	MSI-S	50	wt	N/A
		cecum	/	85–90	100	100	100	0	MSI-H	55	N/A	22
III-2	no	/	blood	0	N/A	N/A	N/A	N/A	N/A	wt	N/A	N/A
II-5	yes	/	blood	0	N/A	N/A	N/A	N/A	MSI-S	50	wt	N/A
		/	lymph node nearby colon	0	N/A	N/A	N/A	N/A	MSI-S	20	N/A	N/A
		/	ovary	0	N/A	N/A	N/A	N/A	MSI-S	50	N/A	N/A
		ileum	/	75	5	100	100	0	MSI-H	20	N/A	N/A
		endometrium (block 1)	/	65	?^1^	100	100	0	MSI-H	50	N/A	N/A
		endometrium (block 2)	/	75	?^1^	100	100	0	MSI-H	50	N/A	N/A
II-6	yes	/	cecum	0	N/A	N/A	N/A	N/A	MSI-S	50	N/A	N/A
		cecum	/	75	100	100	100	0	MSI-H	50	N/A	N/A
I-5	no	/	rectum	0	N/A	N/A	N/A	N/A	MSI-S	wt	N/A	N/A
		rectum	/	70	100	100	100	100	MSI-S	wt	N/A	N/A

N/A—not applicable, IHC—immunohistochemistry, MSI—microsatellite instability, MSI-H—≥3 tested markers were instable, MSI-S—no tested marker was unstable, *MLH1* variant—LRG_216t1:c.2236_2247delCTGCCTGATCTA p.(Leu746_Leu749del), MLPA—Multiplex Ligation dependent Probe Amplification, TMB—tumor mutation burden, ^1^—Immunohistochemical staining of tumor cells was inadequate for assessment of MLH1 expression since MLH1 staining does not show any immunoreactivity in tumor cells nor in internal control (stromal cells, immune cells).

## Supplementary Materials

The following are available online at https://www.mdpi.com/2073-4425/11/3/325/s1, Figure S1: Sanger sequence of novel *MLH1* in-frame deletion c.2236_2247delCTGCCTGATCTA p.(Leu746_Leu749del), Figure S2: Pathohistological features of proband’s mother’s (II-5) tumor - endometrial carcinoma, Figure S3: Pathohistological features of proband’s uncle’s (II-6) tumor - adenocarcinoma of caecum. Table S1: Variants detected in *PMS2* in sample collected from proband’s peripheral blood.
