# Supplementary material for: A Novel Germline MLH1 In-Frame Deletion in a Slovenian Lynch Syndrome Family Associated with Uncommon Isolated PMS2 Loss in Tumor Tissue
Source: Genes (Basel). 2020 Mar 18;11(3):325. doi: 10.3390/genes11030325 (PMC7140785; doi:10.3390/genes11030325)
Supplement: Supplementary file 1 [file genes-11-00325-s001.pdf]

Supplementary Materials

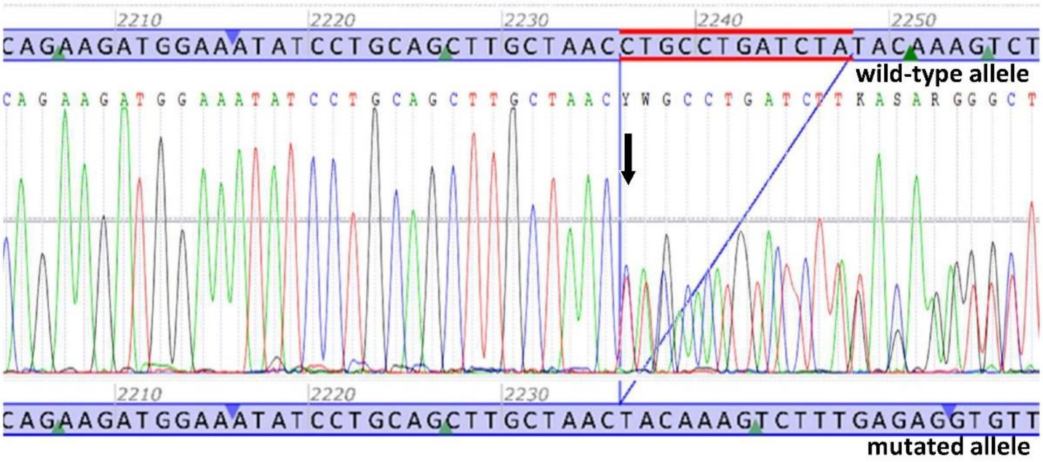

Figure S1. Sanger sequence of novel *MLH1* in-frame deletion c.2236_2247delCTGCCTGATCTA p.(Leu746_Leu749del). Note arrow represents the starting position of deletion.

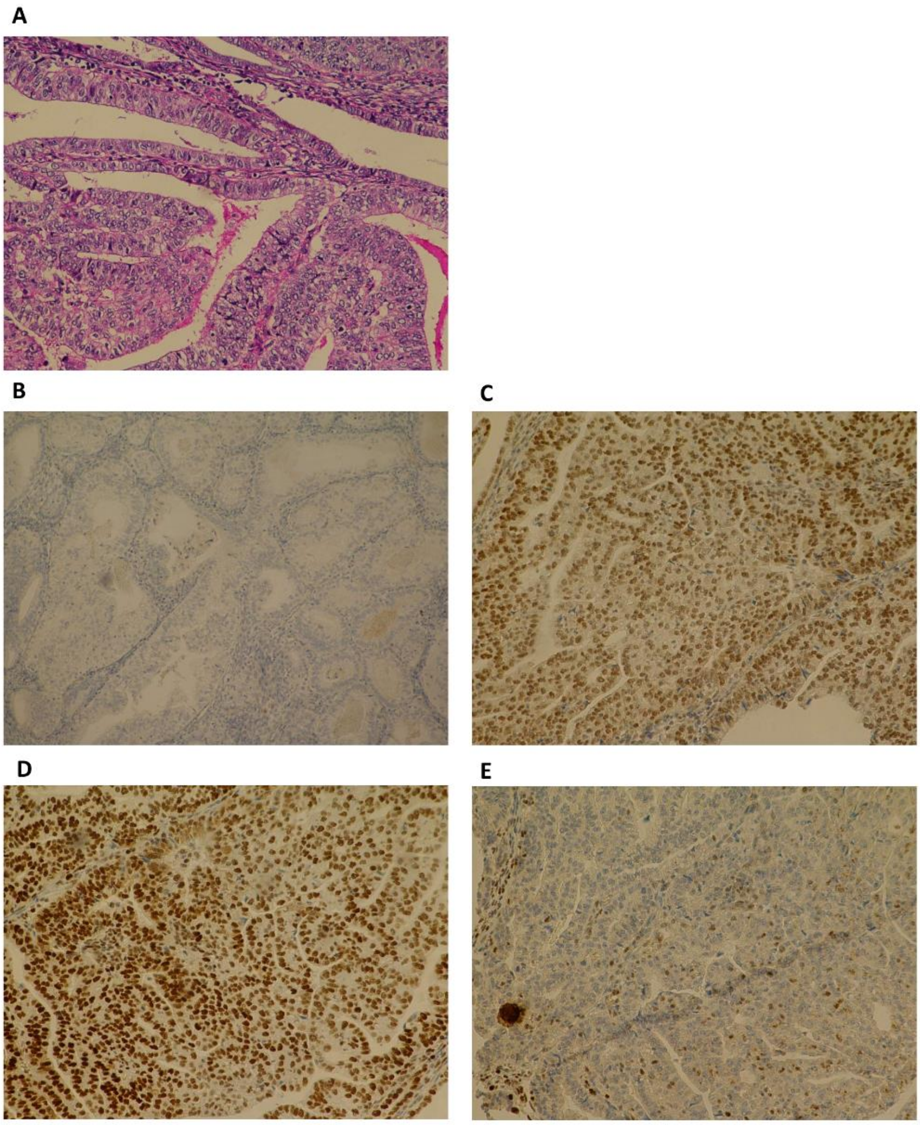

Figure S2. Pathohistological features of proband's mother's (II 5) tumor - endometrial carcinoma. (**A**) Histological analysis (hematoxylin and eosin) revealed a grade 2 endometrial carcinoma consisting of layers of columnar epithelium forming glands. (**B-E**): Immunohistochemical staining of tumor cells was inadequate for assessment of MLH1 expression (**B**) since MLH1 staining does not show any immunoreactivity in tumor cells nor in internal control (stromal cells, immune cells). Retained expression for MSH2 (**C**) and MSH6 (**D**), and loss of expression for PMS2 (**E**) was observed. Note that PMS2 staining was lost in tumor cells, while internal control (stromal cells, immune cells) have intact PMS2 expression.

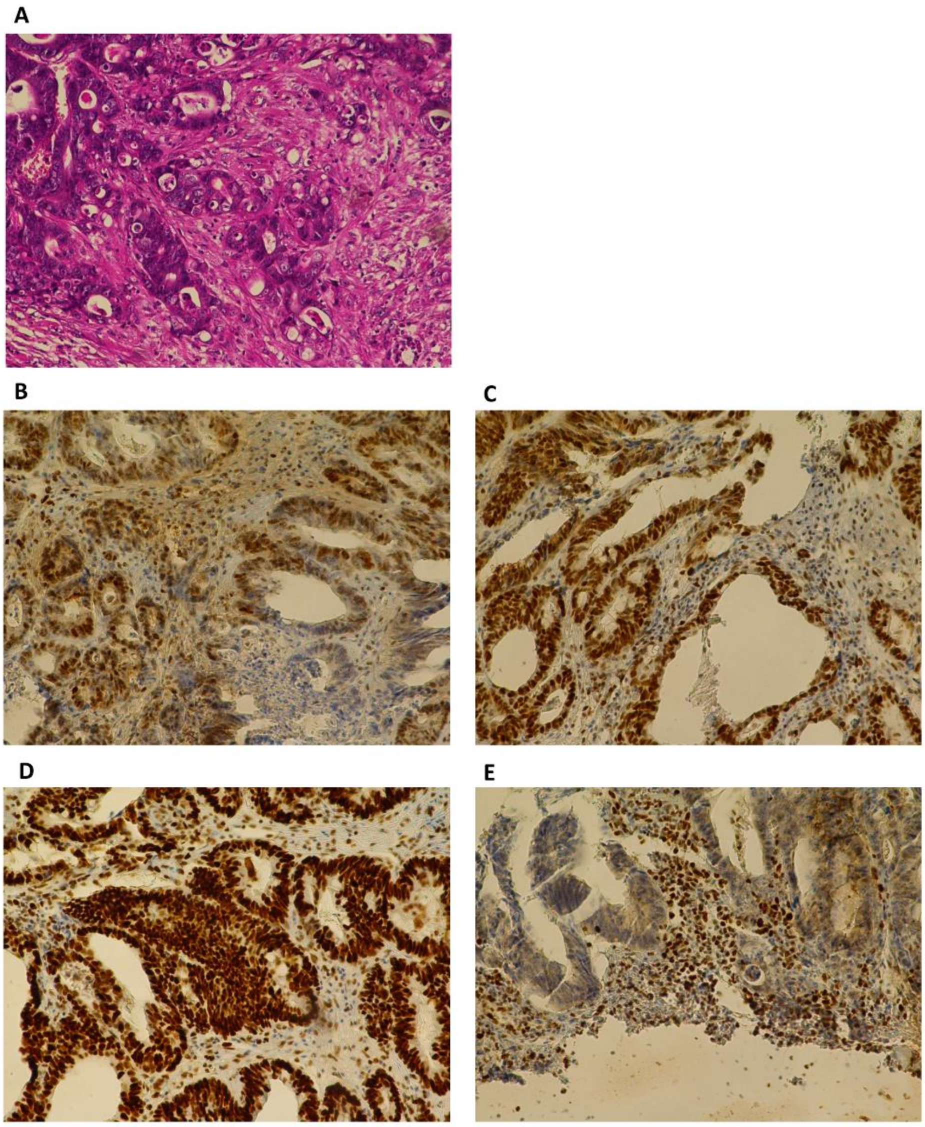

Figure S3. Pathohistological features of proband's uncle's (II 6) tumor - adenocarcinoma of caecum. (**A**) Histological analysis (hematoxylin and eosin) revealed a moderately differentiated adenocarcinoma of caecum. (**B-E)** Immunohistochemical staining of tumor cells showed retained expression for MLH1 (**B**), MSH2 (**C**) and MSH6 (**D**), and loss of expression for PMS2 (**E**). Note that PMS2 staining was lost in tumor cells, while internal control (stromal cells, immune cells) have intact PMS2 expression. Due to the age of the FFPE specimen (more than thirty years), some artifacts and a lesser intensity of staining were noted, particularly in MLH1 and PMS2 stains, but they were deemed still appropriate for assessment. Stains for MSH2 and MSH6 were of sufficient intensity for evaluation.

Table S1. Variants detected in *PMS2* in sample collected from proband's peripheral blood.

Gene	Zygosity	ACMG classification	DNA variant	Predicted effect on the protein (amino acid change)	GnomAD allele frequency in all populations
PMS2	heterozygous	Class 1 - benign	c.2570G>C	p.(Gly857Ala)	28.89%
PMS2	heterozygous	Class 1 - benign	c.2006+6G>A	p.?	7.37%
PMS2	heterozygous	Class 1 - benign	c.1454C>A	p.(Thr485Lys)	7.76%
PMS2	homozygous	Class 1 - benign	c.780C>G	p.(Ser260=)	80.39%
PMS2	heterozygous	Class 1 - benign	c.288C>T	p.(Ala96=)	7.72%
